# Animal Images Database: Validation of 120 Images for Human-Animal Studies

**DOI:** 10.3390/ani9080475

**Published:** 2019-07-24

**Authors:** Catarina Possidónio, João Graça, Jared Piazza, Marília Prada

**Affiliations:** 1 Instituto Universitário de Lisboa (ISCTE-IUL), CIS-IUL, 1649-026 Lisboa, Portugal; 2 Instituto de Ciências Sociais da Universidade de Lisboa, 1600-189 Lisboa, Portugal; 3 CRC-W-Católica Research Centre for Psychological, Family and Social Wellbeing, 1649-023 Lisboa, Portugal; 4 Department of Psychology, Lancaster University, Lancaster LA1 4YW, UK

**Keywords:** human-animal relations, normative data, subjective ratings, diet, meat consumption, animal images

## Abstract

**Simple Summary:**

With the general goal of increasing knowledge about how individuals perceive and evaluate different animals, we provide normative data on an extensive set of open-source animal images, spanning a total of 12 biological categories (e.g., mammals, insects, reptiles, arachnids), on 11 evaluative dimensions (e.g., valence, cuteness, capacity to think, acceptability to kill for human consumption). We found that animal evaluations were affected by individual characteristics of the perceiver, particularly gender, diet and companion animal ownership. Moral attitudes towards animals were predominantly predicted by ratings of cuteness, edibility, capacity to feel and familiarity. We hope this free resource may help advance research into the many different ways we relate to animals.

**Abstract:**

There has been increasing interest in the study of human-animal relations. This contrasts with the lack of normative resources and materials for research purposes. We present subjective norms for a set of 120 open-source colour images of animals spanning a total of 12 biological categories (e.g., mammals, insects, reptiles, arachnids). Participants (*N* = 509, 55.2% female, *M*_Age_ = 28.05, *SD* = 9.84) were asked to evaluate a randomly selected sub-set of 12 animals on valence, arousal, familiarity, cuteness, dangerousness, edibility, similarity to humans, capacity to think, capacity to feel, acceptability to kill for human consumption and feelings of care and protection. Animal evaluations were affected by individual characteristics of the perceiver, particularly gender, diet and companion animal ownership. Moral attitudes towards animals were predominantly predicted by ratings of cuteness, edibility, capacity to feel and familiarity. The Animal Images Database (Animal.ID) is the largest open-source database of rated images of animals; the stimuli set and item-level data are freely available online.

## 1. Introduction

Non-human animals have been ever-present as evolutionary partners with whom humans share the planet. We may care for some species, while perceiving others as inconsequential. The mere thought of eating some animals is disgusting, whereas others are deemed highly appetizing. Animals are treated in varied ways within and across cultures. Some animals are kept as companions, whereas others are used for a myriad of purposes such as work, clothing, entertainment and nourishment. Some are protected, while others are met largely with indifference. People’s perceptions, emotions and attitudes toward non-human animals tend to be diverse, complex and sometimes even paradoxical [1,2]. Researchers have recently been interested in better understanding this complexity, since the way we think about animals and their features has direct consequences for how we treat them.

Interest in addressing such questions is ever increasing among both lay and academic audiences. Progress on these topics will depend partly on the availability of resources for reliable comparisons of results across studies and samples. The publication of norms for sets of stimuli is increasingly important for the scientific community. Particularly, in areas such as affective and moral science, large, diverse and systematically validated stimulus sets have contributed for considerable scientific progress. For instance, it is possible to find normative data for a wide range of visual stimuli, including symbols (e.g., [3,4]), emojis (e.g., [5]), as well as real life pictures (e.g., [6]). Some of the pictures datasets are content-specific (e.g., food [7,8,9]; or faces [10,11]). To our knowledge and in spite of increasing research interest in topics which address our relations with animals, there are no databases providing a comprehensive and systematic depiction of a wide range of different animal species. A recent study produced ratings for fear, disgust and aesthetic preferences for a set of standardized images of reptiles [12] but no other animals were included.

Researchers doing work on use of animals as food often include photos of animals in their materials to study psychological processes such as meat-animal dissociation [13,14], appetite for animal products (e.g., [15,16,17]) and mind attribution (e.g., [18,19,20]). Likewise, researchers doing work on the psychology of animal treatment often make use of animal images in their experimental designs (e.g., [16,21,22,23]). Arguably, studies of this sort could benefit greatly from having access to a versatile set of animal images.

To address this limitation, the present study presents 120 open-source colour animal pictures, from several biological classes, with normative ratings in multiple domains, including affective dimensions (valence, arousal), animal characteristics (both physical and psychological) and attitudes related to the treatment of animals.

### 1.1. Diversity in How We Perceive and Categorize Animals

Humans tend to classify and evaluate non-human animals differently, based on several factors. One criterion is to consider their biological classification. This type of classification aims to describe species, their genetic variability and relationships between animals, according to a predetermined system [24]. Biological classification has been shown to impact on the treatment of animals. Humans have more positive attitudes toward mammals and birds than species from other classifications [25]. For example, humans are more likely to support conservation efforts directed at birds and mammals than for reptiles and invertebrates [25,26,27]. Another criterion is to classify animals based on their utility or relationship to humans, with certain categorizations (e.g., companions) generating more positive outcomes than others (e.g., pests, see Reference [28]).

Characteristics of the perceiver also play a role in how we evaluate different animals. Such characteristics include gender, age, residential area (urban vs. rural), dietary patterns (e.g., meat avoidance) and childhood contact with animals. For instance, compared to men, women tend to report more positive and compassionate attitudes toward animals [16,29,30] and are more concerned with animal protection [31,32]. In contrast, meat consumption is often related to masculinity (e.g., [33,34]) and men are usually more attached to meat [35]. Furthermore, compared to women, men are more likely to endorse “speciesist” attitudes such as believing that humans have the right to use or control animals because of their inferior moral standing [36]. Diet is an important moderator of animal-directed attitudes and judgments, as is childhood experiences with animals. Omnivores tend to attribute animals with less mental and emotional complexity than do vegetarians [37] and several studies have found links between forming an attachment to pets in childhood and levels of empathic concern for animals in adulthood [38,39,40].

### 1.2. Relevant Evaluative Dimensions in How Humans Perceive Non-Human Animals

The current work aims to provide an extensive set of animal images—the Animal Images Database or Animal.ID—to researchers working in the field of human-animal interactions. We thought an extensive database of animal images, with established ratings on a range of perceptual and evaluative characteristics, would be of particular value to researchers interested in lay judgments of animals of various sorts. To this end, we sought to identify a set of evaluative dimensions that researchers would benefit from, based on previous studies within the field. This review of the literature led us to 11 dimensions, which we describe more fully below.

#### 1.2.1. Valence

Valence can be described as the inherent positivity-negativity/ attractiveness-aversiveness of a stimulus (e.g., [41]). It is one of the most relevant dimensions of affect [42]. The valence generated by an animal is likely to influence our emotional and behavioural responses toward them. For instance, animals such as spiders and snakes are generally negatively evaluated and often evoke an avoidance response [43,44]. In contrast, companion animals, such as dogs and cats, generally induce more positive evaluations and attachments [25,45,46].

#### 1.2.2. Arousal

This measure is defined as the level of activation or, conversely, emotional calmness, a person experiences [47]. Similar to valence, arousal is a central dimension of affect. Research examining how individuals perceive the arousal of different animals is scarce (for an exception, see Reference [48] and interplay between arousal and valence is complex. Some studies have shown that negatively evaluated animals (e.g., spiders, snakes) elicit high arousing levels [44]. On the other hand, valence ratings and levels of arousal can positively correlate. For example, studies have shown that pictures representing species with more infantile characteristics generate more positive valence and also higher arousal than pictures of species without these characteristics [49]. Thus, it is important to measure valence and arousal independently to better understand how people evaluate different animals along these dimensions and how these dimensions can interact within such evaluations.

#### 1.2.3. Familiarity

This measure refers to the level or frequency of contact one has with a particular animal in daily life, whether physical or virtual (e.g., via media). Companion animals such as dogs are highly familiar [50], as are many domesticated animals used for food (e.g., pigs) and some well-known wild animals (e.g., lions). Nonetheless, many animals may vary widely in their familiarity. Thus, familiarity may be a highly individualized dimension. Evaluations of animals along this dimension are likely to depend on individual characteristics that would modulate one’s contact with animals. For instance, it is likely that a person who lives in a rural area encounters chickens and cows more often that someone from an urban area.

#### 1.2.4. Similarity to Humans

Animals differ in the extent to which they are perceived to share characteristics in common with humans. Research suggests that similarity influences the evaluation of animals in a number of important ways: from an early age, people tend to prefer animals they consider to be more similar to humans, for example, preferring mammals to birds, birds to herptiles (reptiles, amphibians) and herptiles to invertebrates [21,26,51]. People also report being more likely to “save” animals that share similarities with humans (e.g., [25,52]). For example, a study examining lay decisions about which endangered animals should be prioritized within animal conservation efforts in Australia revealed that ‘similarity to humans’ was the predominate predictor of how participants prioritized different species [53].

#### 1.2.5. Dangerousness

The perceived threat from a species is a relevant measure that affects our perception and treatment of animals [22]. Animals such as snakes, spiders and bats are commonly evaluated as dangerous and associated with phobias and feelings of disgust [54,55,56]. Piazza et al. [22] showed, both with measures and experimental manipulations, that the perceived harmfulness of an animal is predictive of its moral standing, that is, the extent to which it is seen as deserving rights and protections. Importantly, the authors found that the perceived harmfulness of an animal influences its moral standing independent of other morally relevant characteristics, such as the animal’s perceived intelligence or emotional sensitivity.

#### 1.2.6. Cuteness

Cuteness is a perceptual judgment linked to the possession of physical qualities characteristic of infants of many species. These qualities include a large, rounded head, chubby cheeks, and, proportionate to head size, small nose and big eyes—the so-called “baby schema” [57]. Judgments of “cuteness” have been shown to motivate propensities to respond to the subject with care and affection [57,58,59]. An animal’s relative cuteness is an important predictor in how it is treated. Cute features may give certain dogs an advantage to be selected as pets [60]. Baby features have been linked to judgments that an animal is vulnerable and in need of protection. For example, Piazza et al. [16] found that baby farmed animals evoked more feelings of tenderness than their adult counterpart and this increase in tenderness was linked to reduced appetite for meat products associated with the animal. Baby animals appear to be particularly persuasive in promoting animal welfare and intentions to support environmental campaigns [61,62].

#### 1.2.7. Capacity to Think and Capacity to Feel

Capacity to think refers to the animal’s cognitive capacities, such as their capacity to think, imagine and remember [18], whereas capacity to feel refers to the animal’s capacity of feeling and experiencing sensations, such as pleasure and pain. Gray and colleagues [63] subdivide “minds” into two aspects: agency and patiency or higher cognitive abilities (e.g., planning, memory, imagination) and experiential states (e.g., pain, fear, joy). Despite this conceptual distinction, empirical work by Piazza et al. [22] has shown that “thinking” and “feeling” traits tend to be perceived as positively correlated in animals (see also [18]). Critically, when orthogonally manipulated, both dimensions of “mind having” have been found to promote judgments that an animal is worthy of moral consideration [22]. Conversely, when motivated to eat animals, people often deny animals the capacity to think and feel, relative to how these traits are attributed to animals when consumer motivations are removed [18]. As noted earlier, attributions of animal mind are influenced by a number of moderators, including dietary lifestyle [37,64] and familiarity with animals [65]. For example, individuals that live with and care for companion animals report animals of this sort as having richer mental lives than individuals who do not live with companion animals [65].

#### 1.2.8. Edibility

Several factors impact on the perceived edibility of an animal, that is, their judged suitability for human consumption. Cuteness, as previously mentioned, predicts reduced appetite towards meat derived from an animal [16,62]. Likewise, animals ascribed higher degrees of mental capacity are deemed less edible than animals attributed lesser capacities [18,66]. In most Western countries, farmed animals, including cows, pigs and chickens are usually perceived as food sources and highly edible. In contrast, the idea of eating companion animals, such as a dog or cat, is deemed as unthinkable and disgusting [67]. Thus, the way we categorize animals affects their perceived edibility and edibility, in turn, can influence how we attribute characteristics to animals. For example, Bilewicz et al. [37] showed that pigs are denied secondary emotions, compared to dogs, yet this was only true for people who eat meat. Recently, other species have been introduced as sustainable food source alternatives (e.g., crickets, grasshoppers). Additionally, there are some consumers interested in “exotic meat,” including kangaroo or crocodile meat [68,69]. Thus, judgments of edibility are vital for better understanding people’s attitudes towards animals and their use as food products.

#### 1.2.9. Acceptability to Kill for Human Consumption and Feelings of Care and Protection

We included two measures of the judged moral worth of an animal: the extent to which individuals consider it acceptable to harm and kill the animal for human consumption (e.g., food, clothing) and the extent which people desire to care for or protect an animal, items adapted from Piazza et al. [22]. These items, which could be classed as outcome variables, were included to enable us to explore which characteristics, perceived across a vast range of animal targets, are independently predictive of the moral value we place on different animals. Based on the research reviewed earlier [16,18,22,52,53,66], we had reason to believe that the dimensions of human similarity, cuteness, “mind having” (i.e., capacity for thought and feeling), dangerousness and edibility, would each contribute to such moral judgments of animals. However, since all of these dimensions have yet to be tested simultaneously, within a single predictive model, using an extensive set of targets, we reserved judgment with regards to which of the dimensions would emerge as the most predictive and which dimensions might fail to contribute predictive value when controlling for their relationship with other dimensions. Because this aim was exploratory and secondary to our larger goal of validating a large set of animal images, we allowed the zero-order correlations between variables to guide our decision about which variables to include as predictors within our models (see below).

### 1.3. The Current Study

The goals of the present study are threefold: (1) to provide normative ratings for a broad set of animals that can be used by researchers from different areas; (2) to explore how different animals and their characteristics are perceived and evaluated, paying particular attention to which characteristics are independently predictive of people’s moral concern for animals; and (3) to examine how individual differences of the perceiver, including gender, age, diet, living area, companion animal ownership, influence the way animals are perceived and evaluated.

To this end, we developed a database of 120 open-source colour images of animals, spanning a total of 12 biological categories and had each image rated on 11 evaluative dimensions. In selecting our set of animals, we tried to strike a balance between providing a range of biological types, while recognizing that much research, for example, on preferences for animals and their treatment, utilizes mammals more so than other animal categories (e.g., [20,21,22,23]). Thus, we paid particular attention to mammals as a class, while also putting effort into populating other categories often underrepresented in human-animal studies. Furthermore, we also faced pragmatic constraints in our selection process. Certain animal categories, such as mammals and birds, were more abundant within open-source databases than other categories (e.g., arachnids, clitellates). In our analysis, we sought to provide item-level data for each biological category. At the macro-level, we examined associations between the 11 evaluative dimensions across the entire set of animals and contrasted rater evaluations as a function of perceiver characteristics (e.g., gender, diet) and biological classification.

## 2. Materials and Methods

### 2.1. Participants

We aimed to collect at least 500 participants to have a minimum of 50 evaluations per stimulus. Our target sample size was guided by previous studies that have developed normative data and stimulus sets for research purposes (e.g., [4,5,7,9,70]). Five-hundred and seventeen participants completed the survey. After exclusions (see Results section), the final sample included 509 Portuguese participants (55.2% female) aged between 18 and 71 years old (*M*_age_ = 28.05, *SD =* 9.84). More than half of our sample (52.5%) had a higher education degree (Bachelor’s, Master’s or doctorate degree), 41.1% completed secondary education and 6.5% completed primary education. Most participants were students (49.7%) or were employed (42%). The remaining were unemployed (4.7%), retired (0.8%) or reported to have “other” occupational status. Most participants included animals (meat or fish) in their diets (93%), whereas 4.6% followed a vegetarian diet and 2.4% followed a vegan diet. Furthermore, participants reported living in predominantly urban areas (*M =* 5.10, *SD =* 1.93), *t* = 12.87, *p* < 0.001. Although they reported having frequent contact with farmed animals during childhood (*M* = 4.69, *SD* = 2.04), *t* = 7.64, *p* < 0.001, current contact with these animals was less frequent (*M* = 3.09, *SD* = 1.94), *t* = −10.55, *p* < 0.001 (*t-*tests performed against scales midpoint, 4.00). Most participants reported to have had a companion animal during childhood (87.4%), specifically dogs (49.7%), cats (23.3%), fishes (11.3%), hamsters (7.5%) and birds (5.7%). Similarly, most participants reported to currently have a companion animal (72%). Once again, dogs (51.5%) and cats (35.2%) were the most frequent animals, followed by fishes (5.5%) and birds (5.2%). The remaining participants (2.6%) reported having other companion animals (e.g., rabbits, Guinea pigs, horses, goats, chickens, turtles, lizards).

### 2.2. Development of the Stimulus Set

**Figure 1:**
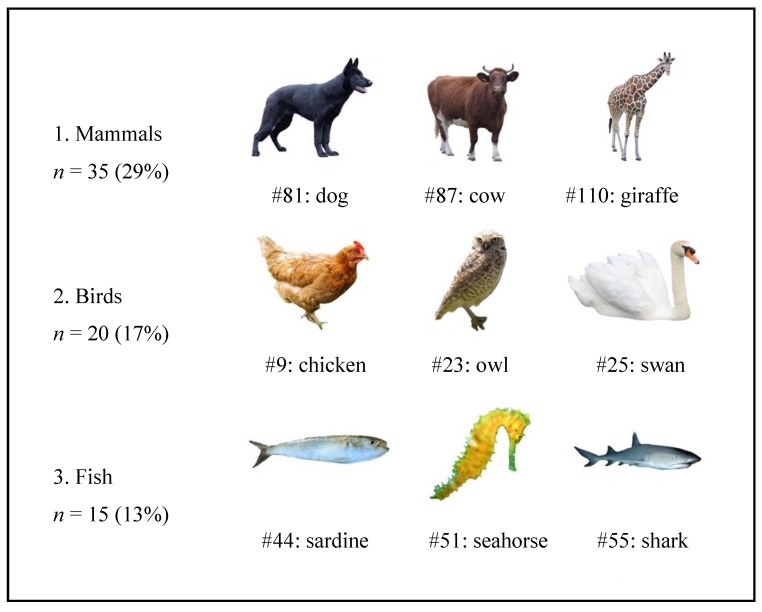
Distribution of the 120 images (*n*; %) according to category and examples of animals included in the stimulus set.

Our database includes 120 open-source colour animal images and is available at https://osf.io/mdpt6/. To develop the stimulus set, animal pictures were retrieved from open-source online databases (e.g., Pixabay; Pexels; Pxhere). The final selection was independently done by four judges, taking the following criteria in consideration: the image was (a) in colour, (b) depicted a single animal and (c) the full body of the animal was visible. Each image depicts a single animal against a white background. The original background of the images was removed to focus attention on the stimulus and to provide images more versatile research uses. Additionally, to standardize stimuli orientation, images were rotated so that, whenever possible, the head of the animal was positioned to the right with a 300 × 225 pixel, PNG format. Furthermore, the images were categorized attending the biological classification of the animal depicted (12 categories, see Figure 1).

### 2.3. Procedure and Measures

Participants were invited via social networking websites and institutional e-mail to collaborate on a web survey aimed at testing stimuli for future research. The language of the survey was in Portuguese. By clicking on a hyperlink, participants were directed to a secure webpage (hosted at Qualtrics©). The opening page informed participants they were taking part in a study on the “perception and evaluation of animal pictures.” They were informed about the study’s expected duration and ethical considerations. After consenting to participate, participants were asked to provide sociodemographic information: age, sex, nationality, educational level and current occupational status. General instructions stated that the task consisted in evaluating each animal on 11 subjective dimensions using 7-point rating scales (for detailed instructions for each dimension, see Table 1). Participants were informed that there were no right or wrong answers. A practice trial was included to familiarize participants with the task (the practice stimulus was not included in the final set of images). To prevent fatigue, participants were asked to rate a subset of 12 animal pictures which were randomly selected from the 120 available. Each trial corresponded to the evaluation of one photograph, with the image centred on the page and the rating scales below it. Upon completion of the evaluative task, we asked participants to indicate their previous and current area of residence (1 = *Predominantly rural*; 7 = *Predominantly urban*), as well as their previous and current contact with both companion animals and farmed animals, using 7-point rating scale (1 = *Not often*; 7 = *Very often*). Next, we asked about participants’ diet and consumption frequency of foods (e.g., red meat, white meat, fruits and vegetables). Finally, participants were thanked and offered the possibility to register for a raffle to win a tablet as compensation for their participation.

### 2.4. Statistical Analysis

Statistical analyses were performed using IBM SPSS Statistics v.23. Zero-order correlations were calculated to examine relationships between the evaluative dimensions. Analysis of variance was used to test for differences in evaluations as a function of our demographic variables (e.g., gender, age, diet) and biological classification. When conducting follow-up comparisons, we used Tukey’s HSD tests to contrast evaluations based on diet and Bonferroni correction to contrast evaluations for comparisons involving biological classification. Linear regressions were conducted to examine which dimensions best predicted our moral outcome variables.

## 3. Results

Only participants that completed the animal pictures evaluation task were retained for analyses (*N* = 517). A preliminary data analysis showed evidence of systematic responding (i.e., same value of the response scales used in 80% of the ratings), which lead to the exclusion of eight participants (final sample = 509). Results reported a small percentage of outliers (1.33%—identified considering the criterion of 2.50 *SD*s above or below the mean evaluation of each stimulus in a given dimension). Therefore, no further responses were excluded.

Each photograph was evaluated, on average, by 51 participants (range: min. = 42, max. = 60), which is within an acceptable range for developing normative data (e.g., [4,5,7,9,70]). Item-level data (means, *SD*s and confidence intervals for each stimulus across evaluative dimensions) is available as Supplementary Data at https://osf.io/mdpt6/.

### 3.1. Frequency Distribution

**Figure 2:**
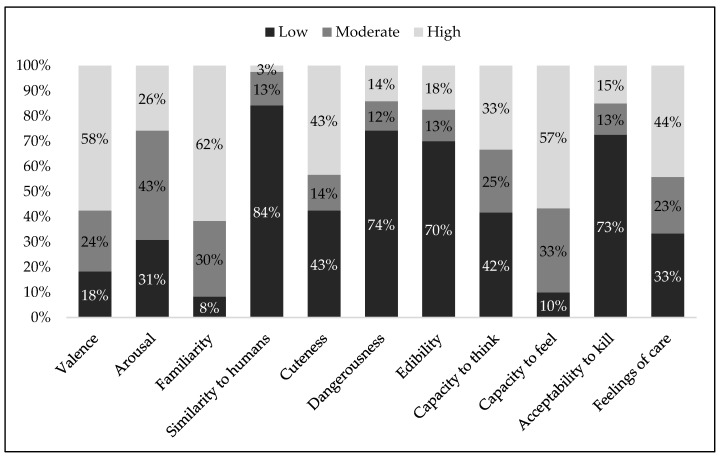
Distribution of animal images with low, medium and high rating on each evaluative dimension.

We computed means, standard deviations and confidence intervals for each image on each dimension (see Supplementary Data). Based on the confidence interval, images were categorized as low, moderate or high on each dimension. Animal images were categorized as moderate when the confidence interval included the response scale midpoint of 4.00; as low when the upper bound of the confidence interval was below the scale midpoint; and as high when the lower bound of the confidence interval was above the scale midpoint (for similar procedure, see References [4,11,70]. Figure 2 represents the frequency distribution of animal images, rated low, moderate and high, on each of the 11 rated dimensions (i.e., valence, arousal, etc.). In the text below, we also provide some examples of animals that fell within each grouping (low, moderate, high).

As shown in Figure 2, most animals were rated as positive (58%, e.g., dolphin, penguin), as familiar (62%, e.g., cat, rabbit) and as having high capacity to feel (57%, e.g., gorilla, dolphin). A smaller percentage was categorized as negative (18%, e.g., mosquito, fly) or with moderate valence (24%, e.g., cricket, squid), low on familiarity (8%, e.g., leech, krill) or as moderately familiar (30%, e.g., frog, shrimp) and has having lower (10%, e.g., clam, sea snail) or moderate capacity to feel (33%, e.g., scorpion, seahorse). Furthermore, most animals were rated as low on the remaining dimensions, namely similarity to humans (84%, e.g., sea snail, fly), dangerousness (74%, e.g., clam, snail), edibility (70%, e.g., fly, mosquito, koala), acceptability to kill for human consumption (73%, lion, dog). A small percentage was perceived as highly (3%, e.g., gorilla, chimp) or moderately (13%, e.g., elephant, horse) similar to humans, highly (14%, e.g., tiger, crocodile) or moderately dangerous (12%, e.g., kangaroo, panda), highly (18%, e.g., sardine, sea bass) or moderately (13%, e.g., rabbit, guppy) edible and highly (15%, e.g., codfish, lobster) or moderately (13%, e.g., goose, squid) acceptable to kill for human consumption.

In the remaining dimensions, the images were distributed across the three levels. For example, ratings for the cuteness dimension show that animals were judged as low (43%, e.g., fly, cockroach) or high (43%, e.g., dolphin, cat) on this dimension, with a smaller percentage of animals categorized a moderately cute (14%, e.g., seagull, cow). Most animals received high feelings of care (44%, e.g., cat, dolphin), with similar distributions across low (33%, e.g., mosquito, fly) and a smaller percentage rated as moderate (23%, e.g., turkey, frog) on feelings of care. Concerning capacity to think, animals were evenly categorized across low (42%, sea snail, mussel, clam), moderate (25%, e.g., swan, chameleon) and high capacities (33%, dolphin, chimp). Moreover, most animals received moderate arousal scores (43%, e.g., ladybug, pig), with similar distributions across low (31%, e.g., sea snail, woodlouse) and high arousal (26%, e.g., lion, cat).

### 3.2. Correlations between the Evaluative Dimensions

Correlations between the evaluative dimensions can be seen in Table 2. Taking the strength of the correlation as our criterion [72], we only discuss in the text correlations that were at least moderate (Pearson’s *r* ≥ 0.40). For example, valence was positively correlated with arousal, familiarity, cuteness, feelings of care, capacity to feel and capacity to think ratings. Arousal was positively correlated with familiarity, cuteness, feelings of care, capacity to feel and capacity to think. We observed moderate positive correlations between ratings of similarity to humans and capacity to think. Cuteness ratings were positively correlated with feelings of care, capacity to feel and capacity to think. Edibility was strongly and positively correlated with moral concern, such that animals perceived as edible were also deemed as more acceptable to kill for human consumption. The ratings for feelings of care were positively correlated with both capacity to feel and to think. Capacity to think and to feel were also strongly, positively correlated.

### 3.3. Differences in Ratings: Individual Characteristics

Table 3 presents a summary of the mean evaluations across dimensions, for the entire sample and separately by gender. Overall, participants evaluated the animal images above the scale midpoint in valence, familiarity, feelings of care and capacity to feel, all *p-*values ≤ 0.004 and below the scale midpoint in the remaining measures, all *p-*values ≤ 0.043. Mean ratings for cuteness, *p* = 0.609 and capacity to think, *p* = 0.583, did not differ significantly from scale midpoint.

#### 3.3.1. Gender

Differences according to participants’ gender in these overall evaluations were only found for a few dimensions. As shown in Table 3, women (vs. men) evaluated the animals as more familiar, *t*(507) = −2.11, *p =* 0.035, Cohen’s *d* = 0.18, more capable to feel, *t*(507) = −2.78, *p =* 0.006, Cohen’s *d* = 0.24, less edible, *t*(454.229) = 6.17, *p <* 0.001, Cohen’s *d* = 0.55 and less acceptable to kill for human consumption, *t*(444.681) = 5.47, *p <* 0.001, Cohen’s *d* = 0.50.

#### 3.3.2. Age

Overall, animal ratings did not differ much according to participants’ age. However, results showed that age correlated with valence, *r* = 0.15, *p* = 0.001, cuteness, *r* = 0.13, *p* = 0.003 and feelings of care ratings, *r* = 0.11, *p* = 0.014. Specifically, the older the participant, the higher were the valence, cuteness and feelings of care ratings of animals.

#### 3.3.3. Diet

To examine the impact of dietary habits on animal evaluation we recoded the type of diet reported by the participant according to the meat ingestion: omnivores (i.e., people who included meat in their diets in an unrestricted manner; 81.4%), restricted omnivores (i.e., pescatarian and flexitarian diets; 11.4%) and meat avoiders (i.e., vegetarians or vegans; 7.2%). As expected, results showed significant mean differences on most of the ratings, except familiarity, as a function of diet (see Table 4). Step-wise differences generally emerged, with the largest mean differences observed between omnivores and meat avoiders, with values for restricted omnivores generally falling between the two. Meat avoiders evaluated animals higher on valence, arousal, cuteness, similarity to humans, capacity to feel, capacity to think, feelings of care and with lower edibility and acceptability to kill, in comparison with omnivores and meat reducers, all *p-*values ≤ 0.022, *η*_p_^2^ = 0.03 to 0.09. Meat avoiders also evaluated animals as less dangerous than omnivores, *p* < 0.001, *η*_p_^2^ = 0.03. Furthermore, compared to omnivores, meat reducers evaluated animals as less acceptable to kill for human consumption, *p* < 0.001, *η*_p_^2^ = 0.09 and displayed higher feelings of care for them, *p* = 0.022, *η*_p_^2^ = 0.09.

#### 3.3.4. Living Area

Overall, animal ratings did not differ much according to participants’ living area. The only exception was for ratings of animals’ capacity to feel, *r* = 0.09, *p* = 0.057, with marginally greater attributed capacity to feel to animals among those participants from urban areas.

#### 3.3.5. Companion Animal Ownership

Results showed differences between participants who currently had a companion animal and participants who had not in animal ratings. Particularly, participants who reported currently owning (vs. not owning) a companion animal rated animals higher in the following dimensions: valence, *t*(313.558) = −2.37, *p* = 0.019, Cohen’s *d* = 0.22, arousal, *t*(506) = −2.79, *p* = 0.031, Cohen’s *d* = 0.28, cuteness, *t*(506) = −3.10, *p* = 0.002, Cohen’s *d* = 0.31, feelings of care, *t*(506) = −3.38, *p* = 0.001, Cohen’s *d* = 0.34, capacity to feel, *t*(282.615) = −2.81, *p* = 0.005, Cohen’s *d* = 0.27, capacity to think, *t*(308.101) = −3.51, *p* = 0.001, Cohen’s *d* = 0.33. Moreover, those who currently had a companion animal also rated animals lower in dangerousness, *t*(506) = 2.90, *p* = 0.004, Cohen’s *d* = 0.28, edibility, *t*(506) = 2.57, *p* = 0.011, Cohen’s *d* = 0.25 and acceptability to kill for human consumption, *t*(506) = 2.84, *p* = 0.005, Cohen’s *d* = 0.28 (see Table 5).

Similarly, results also showed differences in animal ratings between participants who had a companion animal during childhood and participants who had not. Particularly, participants who reported to own a companion animal during childhood rated animals higher in the following dimensions: valence, *t*(506) = −2.32, *p* = 0.021, Cohen’s *d* = 0.33, arousal, *t*(506) = −2.61, *p* = 0.009, Cohen’s *d* = 0.35, cuteness, *t*(506) = −3.03, *p* = 0.003, Cohen’s *d* = 0.43, feelings of care, *t*(506) = −2.96, *p* = 0.003, Cohen’s *d* = 0.40 and capacity to think, *t*(506) = −2.28, *p* = 0.023, Cohen’s *d* = 0.30 (see Table 6).

### 3.4. Differences in Ratings: Animals’ Biological Categories

We categorized the animal images according to their biological category and compared mean ratings across each category (see Figure 1), using a repeated measures ANOVA for each evaluative dimension (with Huynh-Feldt correction as sphericity assumption was not verified). Based on post hoc comparisons with Bonferroni correction, we identified categories with the highest and lowest score in each dimension (see Table 7).

Overall, ratings were highly affected by animal categories in all dimensions (all *p-*values < 0.001, 0.34 < η_p_^2^ < 0.75; see Table 7). Mammals were rated as the most positive animal category (all other comparisons with mammals, *p-*values < 0.001). Arachnids were the most negative category, though the means were not significantly different in comparison to clitellates (all other comparisons with arachnids, *p-*values ≤ 0.003). Mammals were also the most arousing category and clitellates were rated as the least arousing category, all *p-*values < 0.001. Likewise, mammals were rated the most familiar category and clitellates were the least familiar category, all *p-*values ≤ 0.001. The same pattern was observed for cuteness, such that mammals were rated as the cutest category, all *p-*values < 0.001; and clitellates were the least cute, though not significantly less cute than arachnids and bivalves, all *p-*values ≥ 0.628 (all other comparisons with clitellates, *p-*values ≤ 0.001). Expectedly, mammals were the category rated as more similar to humans from all the categories, all *p-*values < 0.001. Bivalves were the category with lowest similarity to humans.

Arachnids were rated the most dangerous animal category, all *p-*values < 0.001. Bivalves were rated the least dangerous, along with gastropods (all other comparisons with bivalves and gastropods, all *p-*values ≤ 0.001). Bivalves were the most edible category, though not significantly more edible than cephalopods, fish and malacostrans (all other comparisons with bivalves, *p-*values ≤ 0.033). Clitellates were the category with the lowest edibility.

Mammals were attributed, by far, the highest capacity to think (all comparisons with mammals, *p-*values < 0.001) and the highest capacity to feel, all *p-*values < 0.001. The lowest thinking capacity was attributed to bivalves, clitellates, gastropods, insects and malacostrans. The lowest capacity to feel was attributed to bivalves and clitellates, all *p-*values ≤ 0.037. Bivalves were rated the most acceptable category to kill for human consumption, though not significantly more acceptable to kill than cephalopods, fish, gastropods and malacostrans, *p-*values ≥ 0.331 (all other comparisons with bivalves, *p-*values < 0.001). Amphibians were rated the least acceptable to kill for human consumption. Finally, mammals were the category that elicited the highest feelings of care and protection, all *p-*values < 0.001. Arachnids received the lowest feelings of care and protection, along with bivalves and clitellates (all other comparisons with bivalves, all *p-*values ≤ 0.016).

### 3.5. Evaluative Dimensions Predicting Moral Attitudes towards Animals

Two multiple linear regression were conducted to predict our two moral outcome variables: (1) acceptability to kill form human consumption and (2) feelings of care and protection. Using the raw correlations to guide us, the following predictor variables were included in the model because they correlated to a significant degree with at least one of the outcome variables: familiarity, cuteness, dangerousness, edibility, similarity to humans, capacity to feel and capacity to think. Multicollinearity analysis showed no concerns on this assumption (*Tolerance* = 0.45 to 0.92, *Variance Inflation Factor* = 1.09 to 2.24).

For acceptability to kill form human consumption, results showed a significant regression equation, *F*(7,501) = 175.37, *p* < 0.001, with the predictor variables explaining 71.0% of acceptability to kill for human consumption. Familiarity (*β* = 0.054), cuteness (*β* = −0.110), edibility (*β* = 0.813) and capacity to feel (*β* = −0.072) contributed at statistically significant levels to the prediction of acceptability to kill, all *p-*values ≤ 0.045. The remaining variables did not statistically contribute to the model (*β* ≤ −0.046), all *p-*values ≥ 0.196. Regarding feelings of care and protection, results also showed a significant regression equation, *F*(7,501) = 88.32, *p* < 0.001, with the predictor variables explaining 55.2% of feelings of care and protection. Similar to the first model, cuteness (*β* = 0.602), edibility (*β* = −0.087) and capacity to feel (*β* = 0.082) made statistically significant contributions to the prediction of feelings of care, all *p-*values ≤ 0.050. The remaining variables did not significantly contribute to the model (*β* ≤ 0.083), all *p-*values ≥ 0.063.

## 4. Discussion

The present study aimed to increase knowledge on how individuals perceive and evaluate different animals and provide normative data on an extensive set of animal images. The publication of norms for sets of stimuli is important for the advancement of scientific research. Here we provide ready-to-use experimental materials for reliable comparisons of animal evaluations across a range of key demographic variables (e.g., gender, diet, animal ownership) and biological classification. The Animal.ID is the first database that includes a vast array of species along with standardized evaluative dimensions beyond valence and arousal. Importantly, this new database of images is completely open-source. Despite it being possible to find a plethora of animal images online, most of them are copyrighted or require payment, which is a barrier to many researchers. Given the recent increase of interest in the study of human-animal relationships and the psychology of animal treatment (for recent reviews, see References [71,73,74]), we believe that the time is ripe to offer the field a set of validated resources to help support progress in this area.

The present database includes 120 open-source colour pictures depicting animals from 12 biological categories, with normative data on 11 measures. Many of the subjective dimensions assessed correlated at low to moderate levels. Valence was correlated with familiarity, which is in line with previous studies supporting the idea that we have more positive attitudes toward familiar stimuli [75]. Our findings also showed that animal category highly affected ratings. Mammals and birds were the biological categories perceived as most positive, cute and familiar. Across all species, cuter animals were perceived as less dangerous, more capable to feel and to think, less acceptable to kill for human consumption and evoked more feelings of care and protection. The link between cuteness and moral concern extends past work by Piazza et al. [16], which focused on evaluations of farmed animals. The relationship between cuteness and mind attribution is consistent with Sherman and Haidt’s [76] mentalizing theory of cuteness, which asserts that cute features enhance social engagement with and mentalizing of the target agent. Our results are also consistent more broadly with the literature on baby schemas, which tends to find more positive outcomes and evaluations of targets, human and nonhuman, that display high levels of cute features [17,58,59,77]. It is interesting to notice that cuteness was unrelated with edibility, which, on the surface, is in tension with Piazza et al. [16], who found that images of baby animals reduced appetite for meat, particularly for women. Since Piazza et al. focused mainly on farmed animals, it may be that the negative relationship between cuteness and edibility does not extend beyond this sub-group of animals.

Our results were furthermore in line with research that suggests that animals perceived to be similar to humans are evaluated more positively (e.g., [21,25,26]). Accordingly, mammals (e.g., gorilla, dolphin) were perceived as most similar to humans and likewise were perceived as the most arousing, cuter, thinking, emotional capable and evoked the highest feelings of care and protection from among all of the animal categories. Such findings parallel those within the literature on inter-group relations. Humans tend to display more favourable attitudes towards targets perceived to share similarities with themselves [78,79,80,81,82]. Interestingly and unexpectedly, we found that similarity to humans had no relationship with acceptability to kill for human consumption. This might be because the majority of animals humans typically eat are mammals, while at the same time some of the most cherished and protected animals are mammals. These competing forces might partly explain the absence of a correlation.

Also of note was the finding that dangerousness was positively correlated with judgments of acceptability to kill for human consumption and edibility. This finding might relate to the psychology of hunting. A study that analysed photos of hunters posing with their prey found that levels of achievement satisfaction displayed by the hunter were greater when posing with larger and more dangerous prey [83]. Humans may believe it is more acceptable—and satisfactory—to kill species that are more dangerous, possibly because of the potential threat they pose to humans. Indeed, Piazza et al. [22] found that perceptions of harmfulness tends to reduce judgments that animals deserve moral protections. Independent of this, people may find the meat of dangerous animals to be highly edible by virtue of these animals being common targets of human hunting.

Moreover, animals perceived as more acceptable to kill for human consumption were also evaluated as less capable to think and feel. Findings from previous studies have suggested that categorizing specific groups of animals as food tends to reduce the amount of mind ascribed to them, which in turn helps justify their use for consumption [18,66]. Nonetheless, in our study, edibility was unrelated to capacity to feel and think. This is somewhat in tension with the findings of Bastian et al. [18], who found a moderately sized negative relationship between mind attribution and edibility. There are several differences between our study and theirs, most of all the scope of animals that we used in our study was much larger. Because of this wide scope, the vast majority of the animals in our sample (70%) were rated quite low on edibility, which may has reduced the possibility of observing a relationship between animal edibility and attributions of thought/feelings.

Our two measures of possessing mind—the capacity to feel and think—were positively correlated, which is consistent with past findings (e.g., [18,22]). As argued by Piazza et al. [22], the capacity for thought (e.g., to imagine and remember things, to reflect on the self) may be perceived as essential to having a rich emotional life, such that species with a greater capacity for thought are seen as more capable of sophisticated emotions. Future work, of course, is needed to test this hypothesis more directly.

### 4.1. Individual Differences in the Evaluation of Animals

In line with past research (e.g., [65,84]), the ratings our participants made of animals were influenced by a number of individual differences. We found small to medium effects of gender on animal evaluations. Overall, women reported more favourable perceptions and attitudes toward animals than men. For instance, women considered animals in general less edible, more capable of feeling and less acceptable to kill for human consumption than men. This is consistent with several studies which have shown that women, compared to men, tend to report more positive attitudes toward animals [16,29,30,36], are more concerned with animal protection [31], are less likely to support animal exploitation [85] and are more likely to oppose meat consumption (e.g., [33,34,35,86,87]).

Consistent with past studies (e.g., [37]), we found small to medium effects of diet on evaluations of animals. Meat avoiders in our sample had more positive attitudes towards animals and attributed them greater cognitive and emotional capacities. Additionally, meat avoiders were more aroused by animals, found them cuter, less dangerous and more similar to humans than meat eaters. We found that age and living area played a limited role on the way individuals perceived animals (see also [84]). Our findings indicated that participants living in predominantly urban areas were marginally more likely to experience higher feelings of care and protection toward animals. Older participants were somewhat more positive towards animals and found them overall cuter than younger participants and they expressed greater feelings of care and protection for animals than younger participants as well.

Finally, our data revealed a small positive effect of companion animal ownership on animal evaluations. Specifically, participants that owned a companion animal during childhood and participants that currently owned one, evaluated animals more positively, as cuter, as more capable of thought and expressed more feelings of care for animals than non-owners. Furthermore, participants currently owning a companion animal also evaluated animals as more capable to feel, less dangerous, less edible and less acceptable to kill for human consumption. These results are in line with a recent study showing that participants who reported more contact with animals had more positive attitudes toward animals [32]. These findings support the “pets as ambassadors’” hypothesis, which posits that pet-keeping in childhood may lead to a more general, positive disposition towards animals and environmental conservation later in life [38,88].

### 4.2. Characteristics Predicting Moral Attitudes

Across the entire set of 120 animals, we found that cuter animals, animals judged less edible and more capable of feeling, tended to be treated with greater moral concern, that is, they elicited greater feelings of care and protection and were deemed less acceptable to kill for human consumption. Less familiar animals were also deemed less acceptable to kill for human consumption. Overall, our findings highlight the importance of several factors in predicting the moral attitudes people hold of animals. These factors predominantly related to: (a) the types of minds animals are thought to possess (e.g., [18,84]); (b) the aesthetic qualities an animal possesses relating to a “babylike” appearance (e.g., [16,21]); and (c) the utilitarian motivations people have when relating to animals as consumer products (e.g., [20,51]).

### 4.3. Limitations and Future Directions

Future research into the evaluations people make of animals should of course continue to expound upon our initial investigation here. For example, there may be some benefit to expanding certain aspects of the stimulus set. For instance, the clitellata category only contained two animals—leeches and earthworms. Likewise, we only included one picture of each animal but for some species (e.g., dogs) there may exist great variability in the attitudes people form towards different exemplars within the species. Thus, future research could expand on our database to include more than one exemplar of each animal to provide a larger representation of the category. Furthermore, the images in our photo set depict the entire animal, yet much research suggests that faces—the eyes in particular—are important for emotion recognition and empathic engagement [89,90]. Future work on animals would benefit from developing a normative set of facial images of animals that could be fitted for such face-directed research. Lastly, the present study relied on a convenience sample of Portuguese Internet users. Our sampling methods may have attracted people with a greater interest in and affinity with animals than the average Portuguese person drawn using alternative methods. Future studies should aim to contrast our findings with other, more targeted and diverse samples to assess for convergence and variation when considering different cultural and demographic contexts.

## 5. Conclusions

Animal.ID—found here: https://osf.io/mdpt6/—offers an open-source database of 120 colour images of animals spanning a total of 12 biological categories, each normed on 11 evaluative dimensions. Researchers can use this free resource to help advance knowledge into the many different ways we relate to animals.

## Figures and Tables

**Table 1:** Instructions and scale anchors for each evaluative dimension.

Dimension	Instruction: Indicate to What Extent	Scale
1. Valence (e.g., [8])	… this animal is negative or positive.	1 = *Very negative* to 7 = *Very positive*
2. Arousal (e.g., [8])	… this animal makes you feel activated or excited.	1 = *Does notat allmake me feelactivated*to 7 = *Makes me feel very activated*
3. Familiarity (e.g., [8])	… this animal is familiar.	1 = *Notat allfamiliar* to 7 = *Extremely familiar*
4. Similarity to humans [51,53]	… this animal is similar to humans.	*1 = Notat allsimilar to humans*to 7 = *Extremely similar to humans*
5. Cuteness [16,71]	… this animal is cute.	1 = *Notat allcute* to 7 = *Extremely cute*
6. Dangerousness [22]	… this animal is dangerous or harmful to humans.	1 = *Notat alldangerous* to 7 = *Extremely dangerous*
7. Edibility [18]	… youfind meat from this animaledible.	1 = *Not at alledible*to 7 = *Extremelyedible*
8. Capacity to think [18]	… this animal has cognitive capacities, such as thought, imagination and memory.	1 =*Not at all capable of thinking, imagining, remembering* to 7 = *Very capableof thinking, imagining, remembering*
9. Capacity to feel [18]	… this animal is capable of feeling and experiencing sensations, such as pleasure and pain.	1 = *Notat allcapable of experiencing sensations, such as pleasure and pain.*to 7 = *Very capable of experiencing sensations, such as pleasure and pain.*
10. Acceptability to kill for human consumption [18]	… it isacceptable or unacceptable to kill this animal for human consumption	1 = *Completely unacceptable to kill the animal for human consumption* to 7 = *Completely acceptable to kill the animal for human consumption*.
11. Feelings of care and protection [22]	… you desire to care for or protect this animal.	1 = *I do not at all desire to care for/protect the animal* to 7 = *I strongly desire to care for/protect the animal*

**Table 2:** Correlations between the 11 evaluative dimensions (Pearson’s *r*).

	1	2	3	4	5	6	7	8	9	10
1. Valence	-									
2. Arousal	0.59 ***	-								
3. Familiarity	0.46 ***	0.42 ***	-							
4. Similarity to humans	0.26 ***	0.39 ***	0.14 ***	-						
5. Cuteness	0.67 ***	0.61 ***	0.39 ***	0.39 ***	-					
6. Dangerousness	−0.25 ***	0.01	−0.16 ***	0.10 **	−0.10 **	-				
7. Edibility	−0.04	0.02	0.07	0.08	−0.03	0.23 ***	-			
8. Capacity to feel	0.50 ***	0.48 ***	0.31 ***	0.28 ***	0.45 ***	−0.06	−0.05	-		
9. Capacity to think	0.45 ***	0.52 ***	0.22 ***	0.48 ***	0.48 ***	0.01	−0.07	0.66 ***	-	
10. Acceptability to kill	−0.14 ***	−0.10 **	0.04	0.03	−0.15 ***	0.19 ***	0.83 ***	−0.16 ***	0.17 ***	-
11. Feelings of care	0.63 ***	0.59 ***	0.32 ***	0.34 ***	0.72 ***	−0.14 ***	−0.12 ***	0.44 ***	0.47 ***	0.28 ***

*N* = 509. ** Correlation is significant at the 0.050 level (two-tailed). *** Correlation is significant at the 0.001 level (two-tailed).

**Table 3:** Mean evaluations across dimensions: The full sample and by gender.

	Full Sample (*n* = 509)		Men (*n* = 228)		Women (*n* = 281)	
	*M*	(*SD*)	*M*	*SD*	*M*	*SD*
Valence	4.43 *	(0.96)	4.35 ^a^	(0.93)	4.51 ^a^	(0.99)
Arousal	3.90 *	(1.10)	3.88 ^a^	(1.02)	3.91 ^a^	(1.17)
Familiarity	4.73 *	(1.31)	4.60 ^a^	(1.31)	4.84 ^b^	(1.29)
Similarity to humans	2.51 *	(1.08)	2.54 ^a^	(1.00)	2.49 ^a^	(1.14)
Cuteness	4.02	(1.05)	3.98 ^a^	(0.99)	4.06 ^a^	(1.09)
Dangerousness	3.09 *	(0.82)	3.12 ^a^	(0.82)	3.08 ^a^	(0.82)
Edibility	2.99 *	(1.28)	3.37 ^a^	(1.32)	2.69 ^b^	(1.15)
Capacity to feel	4.76 *	(1.33)	4.58 ^a^	(1.32)	4.90 ^b^	(1.32)
Capacity to think	3.97	(1.31)	3.94 ^a^	(1.17)	3.99 ^a^	(1.42)
Acceptability to kill	2.93 *	(1.36)	3.29 ^a^	(1.44)	2.63 ^b^	(1.21)
Feelings of care	4.17 *	(1.30)	4.15 ^a^	(1.18)	4.18 ^a^	(1.39)

* Different from scale midpoint (i.e., 4). Different superscripts indicate significant differences due to gender, all *p-*values ≤ 0.035 (i.e., values labelled with ^a^ are statistically different from ^b^).

**Table 4:** Mean scores for evaluative dimensions by dietary category (level of meat restriction).

	Omnivores (*n* = 394)		Restricted Omnivores (*n* = 55)		Meat Avoiders (*n* = 35)	
	*M*	*SD*	*M*	*SD*	*M*	*SD*
Valence	4.36 ^a^	(0.86)	4.58 ^a^	(1.11)	5.25 ^b^	(1.09)
Arousal	3.83 ^a^	(1.06)	3.96 ^a^	(0.92)	4.86 ^b^	(1.11)
Familiarity	4.74 ^a^	(1.30)	4.62 ^a^	(1.35)	5.22 ^a^	(1.14)
Similarity to humans	2.41 ^a^	(0.99)	2.54 ^a^	(1.07)	3.20 ^b^	(1.39)
Cuteness	3.94 ^a^	(0.97)	4.11 ^a^	(1.15)	4.69 ^b^	(1.26)
Dangerousness	3.14 ^a^	(0.80)	2.95 ^a,b^	(0.83)	2.58 ^b^	(0.80)
Edibility	3.11 ^a^	(1.25)	2.95 ^a^	(1.19)	2.02 ^b^	(1.29)
Capacity to feel	4.72 ^a^	(1.30)	4.73 ^a^	(1.40)	5.63 ^b^	(1.29)
Capacity to think	3.90 ^a^	(1.29)	3.88 ^a^	(1.14)	4.87 ^b^	(1.43)
Acceptability to kill	3.11 ^a^	(1.35)	2.64 ^b^	(1.00)	1.60 ^c^	(1.06)
Feelings of care	4.02 ^a^	(1.20)	4.49 ^b^	(1.40)	5.42 ^c^	(1.28)

Different superscripts indicate significant differences due to dietary category, all *p-*values ≤ 0.022. Tukey’s HSD tests were used for step-wise comparisons of dietary category.

**Table 5:** Mean scores for evaluative dimensions by current companion animal ownership.

	Current Companion Animal Ownership			
	No (*n* = 142)		Yes (*n* = 366)	
	*M*	*SD*	*M*	*SD*
Valence	4.29 ^a^	(0.82)	4.49 ^a^	(1.01)
Arousal	3.68 ^a^	(1.01)	3.98 ^b^	(1.13)
Familiarity	4.75 ^a^	(1.38)	4.73 ^a^	(1.28)
Similarity to humans	2.48 ^a^	(1.06)	2.52 ^a^	(1.09)
Cuteness	3.79 ^a^	(0.97)	4.11 ^b^	(1.06)
Dangerousness	3.26 ^a^	(0.82)	3.03 ^b^	(0.81)
Edibility	3.22 ^a^	(1.24)	2.90 ^b^	(1.28)
Capacity to feel	4.51 ^a^	(1.23)	4.86 ^b^	(1.36)
Capacity to think	3.67 ^a^	(1.13)	4.08 ^b^	(1.36)
Acceptability to kill	3.20 ^a^	(1.34)	2.82 ^a^	(1.35)
Feelings of care	3.86 ^a^	(1.18)	4.29 ^b^	(1.32)

Different superscripts indicate significant differences due to companion animal ownership, all *p-*values ≤ 0.031.

**Table 6:** Mean scores for evaluative dimensions by companion animal ownership in childhood.

	Companion Animal Ownership in Childhood			
	No (*n* = 64)		Yes (*n* = 444)	
	*M*	*SD*	*M*	*SD*
Valence	4.17 ^a^	(0.84)	4.47 ^b^	(0.98)
Arousal	3.57 ^a^	(1.05)	3.95 ^b^	(1.11)
Familiarity	4.63 ^a^	(1.43)	4.75 ^a^	(1.29)
Similarity to humans	2.45 ^a^	(1.11)	2.52 ^a^	(1.06)
Cuteness	3.65 ^a^	(0.95)	4.08 ^b^	(1.05)
Dangerousness	3.24 ^a^	(0.70)	3.07 ^a^	(0.84)
Edibility	3.19 ^a^	(1.25)	2.96 ^a^	(1.28)
Capacity to feel	4.59 ^a^	(1.22)	4.78 ^a^	(1.35)
Capacity to think	3.62 ^a^	(1.32)	4.02 ^b^	(1.31)
Acceptability to kill	3.18 ^a^	(1.41)	2.89 ^a^	(1.34)
Feelings of care	3.72 ^a^	(1.22)	4.23 ^b^	(1.30)

Different superscripts indicate companion animal ownership differences, all *p-*values ≤ 0.023.

**Table 7:** Means and standard deviations for each evaluative dimension by animal category.

Dimensions	Animal Category												
	Amphibians	Arachnids	Birds	Bivalves	Cephalopods	Clitellates	Gastropods	Insects	Malacostrans	Mammals	Fish	Reptiles	F ***
	*M* (*SD*)	*M* (*SD*)	*M* (*SD*)	*M* (*SD*)	*M* (*SD*)	*M* (*SD*)	*M* (*SD*)	*M* (*SD*)	*M* (*SD*)	*M* (*SD*)	*M* (*SD*)	*M* (*SD*)	
Valence	3.43	2.65	4.73	4.23	4.29	2.76	4.10	3.49	3.99	5.27	4.49	3.95	*F*(8.35,409.29) = 69.35,
	(0.47)	(0.26)	(0.61)	(0.38)	(0.23)	(0.19)	(0.45)	(1.04)	(0.46)	(0.57)	(0.59)	(1.34)	*η*_p_^2^ = 0.59
Arousal	3.33	3.48	4.03	2.76	3.58	2.71	2.84	3.43	3.30	4.75	3.67	4.01	*F*(7.25,355.30) = 24.77,
	(0.47)	(0.30)	(0.39)	(0.13)	(0.40)	(0.13)	(0.47)	(0.52)	(0.52)	(0.54)	(0.41)	(0.38)	*η*_p_^2^ = 0.34
Familiarity	3.84	3.95	5.07	4.18	3.97	2.89	4.44	4.54	3.82	5.41	4.48	4.56	*F*(8.28,405.51) = 28.52,
	(0.46)	(0.79)	(0.55)	(0.79)	(0.91)	(0.64)	(0.98)	(0.61)	(1.16)	(0.59)	(0.69)	(0.55)	*η*_p_^2^ = 0.37
Cuteness	2.94	2.01	4.62	2.30	3.62	1.87	2.71	2.72	2.78	5.50	3.80	3.67	*F*(9.34,457.55) = 125.56,
	(0.82)	(0.07)	(0.94)	(0.34)	(0.44)	(0.04)	(0.71)	(1.27)	(0.39)	(0.79)	(1.07)	(1.28)	*η*_p_^2^ = 0.72
Similarity humans	1.76	1.76	2.56	1.43	2.10	1.50	1.59	1.71	1.70	3.72	2.15	2.17	*F*(8.38,410.61) = 69.38
	(0.21)	(0.11)	(0.38)	(0.15)	(0.24)	(0.00)	(0.26)	(0.24)	(0.07)	(0.86)	(0.22)	(0.37)	*η*_p_^2^ = 0.59
Dangerousness	3.59	5.32	2.81	1.60	3.24	3.01	1.58	2.85	2.83	3.62	2.59	4.03	*F*(6.45,316.15) = 120.40,
	(0.56)	(0.46)	(0.74)	(0.30)	(0.56)	(0.32)	(0.22)	(1.15)	(0.65)	(1.25)	(1.24)	(2.08)	*η*_p_^2^ = 0.71
Edibility	1.85	1.93	3.10	4.38	4.19	1.61	3.66	1.82	4.12	2.71	4.20	2.21	*F*(9.06,443.98) = 103.15,
	(0.25)	(0.09)	(1.09)	(0.91)	(0.79)	(0.04)	(0.97)	(0.35)	(1.63)	(1.27)	(1.45)	(0.71)	*η*_p_^2^ = 0.68
Capacity to think	3.08	3.10	4.37	2.17	3.78	2.52	2.66	2.95	3.07	5.26	3.46	4.16	*F*(7.65,374.80) = 64.25,
	(0.49)	(0.12)	(0.41)	(0.25)	(0.69)	(0.06)	(0.44)	(0.36)	(0.08)	(0.58)	(0.45)	(0.40)	*η*_p_^2^ = 0.57
Capacity to feel	4.17	3.89	5.26	2.85	4.49	3.28	3.60	3.85	3.98	5.83	4.41	4.86	*F*(8.12,397.68) = 49.70,
	(0.42)	(0.31)	(0.27)	(0.31)	(0.50)	(0.0)	(0.50)	(0.42)	(0.15)	(0.43)	(0.39)	(0.59)	*η*_p_^2^ = 0.50
Acceptability kill	2.09	2.52	2.96	4.39	3.94	2.35	3.87	2.38	3.98	2.38	3.83	2.21	*F*(7.88,385.95) = 47.241
	(0.17)	(0.17)	(0.90)	(0.69)	(0.67)	(0.36)	(0.68)	(0.29)	(1.14)	(1.07)	(1.20)	(0.66)	*η*_p_^2^ = 0.49
Feelings of care	3.20	2.44	4.65	2.89	3.72	2.45	3.23	3.0	3.32	5.34	4.07	4.0	*F*(8.79,439.64) = 59.00,
	(0.54)	(0.13)	(0.63)	(0.18)	(0.27)	(0.24)	(0.33)	(0.97)	(0.29)	(0.62)	(0.52)	(1.12)	*η*_p_^2^ = 0.55

*** All *p-*values < 0.001.

## Supplementary Materials

Stimulus set (120 animal images) and item-level data (means, *SD*s and confidence intervals for each stimulus across evaluative dimensions) are available online at https://osf.io/mdpt6/.
